# Editors’ Book Table

**Published:** 1875-01

**Authors:** 


### Editors' Book Table.

[NOTE. — All works reviewed in the pages of the CHICAGO MEDICAL JOURNAL may be found in the extensive stock of W. B. KEEN, COOKE & CO., whose catalogue of Medical Books will be sent to any address upon request.]

THE REPORT OF THE CHICAGO RELIEF AND AID SOCIETY, OF DISBURSEMENTS OF CONTRIBUTIONS FOR THE SUFFERERS BY THE CHICAGO FIRE. Printed for the Chicago Relief and Aid Society, at the Riverside Press. 1874.

This Report constitutes an interesting chapter in the annals of Chicago, and a record of, perhaps, the grandest charitable impulse which ever stirred the sympathies of the world.

Beginning with a brief sketch of the history and topography of the city, and its condition at the time of the fire, to which is superadded a mere mention of the disaster itself, and a somewhat more detailed statement of its results upon the inhabitants, the Report proceeds to the narration of the measures taken for the care of the homeless, the protection of life and property, and the re-establishment of the demoralized municipal government.

The record of the expressions of sympathy from almost every quarter of the habitable globe, is full of interest, and the telegrams and letters by means of which these expressions were conveyed, should be treasured most carefully among the choicest archives of the city, which will serve in the future as reminders of our duty to our neighbors, as stimuli to our charity to those in similar situation.

The Relief and Aid Society have done a noble work, and have done it nobly and faithfully. The amount of administrative ability brought into its organization and continued maintenance, must attract the admiration and merit the respect of the world ; and their "Report" will stand an enduring monument of charity, of fidelity and honor. H.

We welcome with much pleasure to our exchange list a new medical journal from our sister Republic, Mexico. The first and second numbers of the "Repertorio Jalisciense de Medicina y Cirugia Practicas," have just made their appearance, under the dates Oct. 15th and Nov. 1st.

The Journal is a semi-monthly, having a numerous corps of contributors, *i. e.:* Messrs. Agraz, Arce, Arias, Benitez, Camarena, J. M., Clement, Fuentes I., Garciadiego, Perez, Puga, Torres, y Zavala, and is published at the City Guadalajara, in the State of Jalisco.

Its original articles are highly creditable to their authors, and indicative of a high state of medical education in the city of Guadalajara.

We are glad to recognize the good example which is herein placed before us, in the attention given to the subject of Medical Meteorology, a subject destined to reward its diligent cultivators with results which will throw much light upon the laws governing the operation of etiological and epidemiological influences.

In this particular department of science our sanitarians might take some valuable lessons from the carefully arranged meteorological tables of Senor Fuentes, in which not only the thermometric maximum, minimum and mean indication are registered, but also the barometric pressure, variations in atmospheric pressure upon the human body, the hygrometric condition, the rain-fall, the direction of the wind, and the state of the sky, are carefully recorded for collation with the tables of public health and mortality.

Senor Arce reports a case of successful ligation of the primitive carotid artery, necessitated by a gun-shot wound in the neck.

The Editors of the Repertorio are Srs. Clement, Director, S. Garciadiego, N. Puga, to whom we extend a cordial welcome into the field of journalism. H.

THE SCIENCE OF HOMŒOPATHY; OR A CRITICAL AND SYNTHETICAL EXPOSITION OF THE DOCTRINES OF THE HOMŒOPATHIC SCHOOL. By *Chas. J. Hempel, M.D.*, Author and Translator of a number of standard works on Homœopathic Materia Medica and Practice, Honorary Member of several foreign and American Homœopathic Colleges and Societies. Bœricke & Tafel, New York, Philadelphia, Baltimore and San Francisco.

Although regarding this work from a point of view far removed from that occupied by the author, we nevertheless find ourselves in perfect agreement with some of his propositions. For example, in the first part of the work, in which the author discusses the "Philosophy of Homœopathy," and more especially that phase comprising the subject of infinitesimal doses, and "feels likewise called upon to testify to the efficacy of the higher preparations, even the two hundredth, of arsenic," etc., etc., etc., . . "but is not prepared to assert that a lower preparation would not have acted equally well in the cases where these high attenuations produced positive and even brilliant results"—which, in our humble judgment, annihilates the whole theory of potencies.

As an illustration of the permissibility of palliative treatment, on page thirty-three we are told that "a boy nine years old, with consumptive habit, was attacked with racking cough and fever," "had been sick some days," had "burning fever, paroxysms of racking cough, hectic flushes on the cheeks; great dyspnœa; marked dullness over the whole chest, especially over the upper lobes of the lungs, no respiratory murmur perceptible in the upper half of the chest; pulse 130; bowels constipated; urine scanty and cloudy; slight remission of the symptoms in the forenoon. I treated the child for a whole week with aconite, bryonia, phosphorus, arsenicum," etc., etc., . . . "without being able to effect the least improvement in the symptoms." . . . "At the request of the parents I gave the child a little morphine, in order to afford him some sleep, for which purpose I dissolved half a grain in ten teaspoonsful of water, of which solution the child was to take a teaspoonful (*one-twentieth of a grain*) every half hour until he dropped asleep." "On my next morning visit I was surprised to find the child not only alive, but sleeping sweetly," etc., etc. . . . "In a week after his improvement had commenced he was playing again in the open air,"—after which Homœopathy might exclaim, Save me from my friends!

Later, we are told, "There is a correspondence between the drug-germs in the crust of our planet and the morbid properties inherent in the tissues of the human organism." And again, "In order to cure a pathological disease, we apply to it the drug by which it is typified in nature"—which aphorism, we suppose, embodies the principle, "similia similibus curantur," and recalls vividly the same principles of therapeusis involved in some of the prescriptions contained in old Gervaise Markham, who tells us, "For broken bones, bones out of joint, or any grief in the bones or sinews, oil of swallows was pronounced exceedingly sovereign." The said oil of swallows was to be procured by "pounding twenty live swallows in a mortar," etc.

This looks like the "Doctrine of Signatures," of Oswald Crollius, revived, who taught that as "Walnuts are said to have the perfect signature of the head; the outer crust, or green covering, represents the *pericranium*, or outward skin of the skull, wherein the hair groweth, and therefore salt made of those husks is exceedingly good for wounds in the head. The kernel hath 'the very figure of the brain,' and therefore it is very profitable for the brain, and resists poisons." In what does this differ from the dictum on page 63 of this "Critical and Synthetical Exposition": "the capacities of the specific drug powers in the crust of our planet correspond with specific capacities for disease, and that fully developed drugs correspond with fully developed diseases"?

On the same page we are told that "Vital Force" "is nothing less than the spiritual or living organism."

Of this book, we must be permitted to say that it must be studied in order to be fully appreciated. H.

CYCLOPÆDIA OF PRACTICAL MEDICINE. Edited by *Dr. H. Von Ziemssen*, Professor of Clinical Medicine in Munich, Bavaria. Vol. I. Acute Infectious Diseases. By Prof. Liebermeister, of Tubingen, Prof. Lebert, of Breslau, Dr. Haenisch, of Griefswald, Prof. Heubner, of Leipzig, and Dr. Oertel, of Munich. Translated by R. H. Fitz, M.D., and Charles P. Putnam, M.D., of Boston; Arthur Van Harlingen, M.D., of Philadelphia; James T. Whitaker, M.D., of Cincinnati; Edward W. Schauffler, M.D., of Kansas City; and Francis Delafield, M.D., Horatio Bridge, M.D., J. Haven Emerson, M.D., Thomas E. Satterthwaite, M.D., Lewis A. Stimson, M.D., and Normand Smith, M.D., of New York.
*Albert H. Buck, M.D.*, of New York, editor of American Edition. New York: Wm. Wood & Co. 1874.

The present volume initiates one of the greatest enterprises in the field of medical literature ever undertaken; by the side of which our old Cyclopædia of Forbes & Tweedie, and the more recent "System" of Reynolds, appear dwarfed. In no other land than Germany, perhaps, could the patient research, the plodding industry, the unflagging zeal absolutely necessary to the accomplishment of such a task, have been found; and while awarding to the Germans full credit for the possession of all these qualities, we must at the same time especially commend the boldness of our countrymen, the translators, editor, and publishers, which has prompted them to give the work to the profession on this side of the water, contemporaneously with its appearance on the other—an enterprise which is peculiarly American.

Of the authors of the treatises which constitute the present volume, Prof. Lebert, of Breslau, is from his age and eminence best known upon this side of the water, more especially by means of his researches in the pathogenesis of tubercle and cancer. The name of Liebermeister is perhaps the next in the order of our acquaintance. The rest, however, have not been idle, and though yet young, have made their names familiar sounds to the German medical student.

The present volume comprises the subject of ACUTE INFECTIOUS DISEASES, and contains articles upon Typhoid Fever, both regular and irregular, and upon the Plague, by Liebermeister; upon RELAPSING FEVER, TYPHUS FEVER and CHOLERA, by Lebert; upón YELLOW FEVER, by Haenisch; upon DYSENTERY, by Heubner; and upon EPIDEMIC DIPHTHERIA, by Oertel.

These treatises are voluminous, constituting a volume of seven hundred pages, to which is appended a very complete and satisfactory index.

Liebermeister's treatise upon Typhoid Fever is comprehensive, and the views therein expressed in harmony with those of the best observers in England and in America, to whom, however, he does not refer, basing his conclusions upon evidence drawn almost exclusively from the observations of French and German authorities.

The specific distinctions between typhoid and typhus are well and clearly drawn, and the contagious character of the latter and the non-contagiousness of the former asserted.

The author's conclusions concerning the etiology of the disease are not as satisfactory as is desirable. He rejects the influence of exhalations from excrementitious deposits—sewers, privies, etc.—as efficient causes of the disease *per se*, but assigns this agency to these, and to the imbibition of water contaminated with these matters when containing the specific *typhoid poison*. Here he is guilty of a petitio principii. His theory necessitates the introduction of the specific poison as an essential factor. But from whence does he derive this essential factor? Just here the author leaves us, no closer to the origin of the disease than before, since he will not admit its production by the decomposition of excrementitious matters.

The relations of these causes to the disease are more fully determined, in considering the season of maximum prevalence of typhoid fever, which has been found to differ slightly in different localities. This difference has been found to correspond closely to the differences in the depth of surface soil covering deposits of decomposing organic matters, and also with the period of greatest dryness of atmosphere, and depression of water level in the soil.

These relations above noted do not differ essentially from those apparent to careful observers of the disease in this country. The correspondences of the ranges of temperature with those of the pulse, and the value of these correspondences as prognostic data, are fully set forth, and well illustrated by diagrams.

The description of pathological changes in the intestine, recognized as the essential lesion, and demonstrated by numerous autopsies, is elaborate and graphic. The chapter on prophylaxis is brief but comprehensive, and may be summarized in one sentence: "The points which should receive the greatest attention are drinking water and privies."

The discussion of treatment is very full and complete, including all, or nearly all, to be said upon the subject, one point therein deserving special attention, *i. e.*, the favorable testimony which the author gives to the value of calomel and cold water, and the inutility of iodine. Quinine, veratria, and digitalis, as remedial agents, receive full consideration, but we must be permitted to say that the author's rules for the administration of the latter drug, digitalis, are by no means clear or satisfactory. On page 217 he says, "Digitalis is only to be used in those cases of typhoid fever in which there is no considerable degree of cardiac weakness, where the pulse is not yet extremely frequent, or, at least, is still pretty strong. The rule for its application is just the opposite to what it is in diseases of the heart; now the more frequent the pulse the less the digitalis is indicated. The impending paralysis of the heart is not prevented by the use of this drug, but seems rather to be favored thereby. No special harm is done, in patients with powerful action of the heart, if the administration of a large dose causes nausea and vomiting, of course the medicine must be then stopped." By comparison with the statements of the heart's action on page 82, and its histological changes on page 107, the paragraph quoted above seems somewhat discordant, and in view of the essential relations of cardiac weakness to typhoid fever, and clinical observation of the influence of digitalis in this and similar conditions, seems, to say the least, paradoxical.

There is one other point in the therapeusis, or rather the nutrition of cases of typhoid, to which we direct attention for the purpose of recording a dissent from the author's views, sustained as they are even by so high an authority as Stromeyer, that "Oaten grits is the best thing to give to typhoid fever patients." How such principles of dietetics can be reconciled with the essential pathology of this disease, as ably set forth by our author, in obedience to physiological laws, is incomprehensible. We venture the opinion that the ingestion of farinaceous food under the circumstances here assumed, would almost certainly establish or aggravate diarrhœa, and thus exhaust the patient, directly and indirectly.

Taken as a whole, the article on typhoid fever is interesting and valuable, and will well repay careful study.

The article by the same author, Liebermeister, on the Plague, is interesting historically, although it cannot be said to have much practical value in a portion of the earth where the disease has been hitherto unknown, and will probably never appear. The data furnished by the history of this disease, however, are important in this regard, that, when collated with those supplied by the history of epidemiology at the present time, they supply confirmatory evidence, if that were needed, of the absolutely protective influence of rigid hygienic and sanitary precautions, and strict isolation of the foci of disease. Every additional fact deduced from observation of epidemics in every quarter of the earth, and in every epoch of its history, tends to the truth of the proposition that diseases, like fires, must be fought in front, and that if we cannot cure some of them, we can at least prevent them, which is better.

The second article of the present volume is by Lebert, on Relapsing Fever, the original locality of which he assigns to Great Britain and Ireland.

The author's views of the etiology of relapsing fever may be epitomized by a single sentence, quoted from page 263. "Relapsing fever, since Obermeier's discovery, is certainly one of those infectious diseases for which a protomycetic origin has been most positively established," and "my assistants, Weigert and Buchwald, and myself have examined the blood in all cases of relapsing fever under my care, with the aid of strong immersion lenses, and have arrived at the conclusion that those protomycetes are never absent during the period of invasion and relapse, although they diminish very quickly after defervescence." Without attempting to discuss here the germ theory of disease, we will take the liberty to animadvert upon the superficial mode in which questions of this sort are in this, and in many another instance, decided, in utter defiance of any rules of logic.

That these "protomycetes" were invariably found in the blood of patients suffering from relapsing fever, is an isolated fact which admits of no deductions until collated with evidence which shall comprehend other sides of the subject. Nor is the additional statement, on page 264, that they "never have been found thus far in any other disease," sufficient to establish the author's opinions, for it is highly probable that they may not have been looked for as carefully under other circumstances.

The pathology of the disease, together with that of bilious typhoid, by some considered identical, is well described, as are also the history, geography, topography and symptomatology of the two diseases, comprising much valuable information, which will well repay attentive study.

The same author, Lebert, in his more elaborate article upon Typhus, indicates his adoption of the germ theory of disease in general, as we learn on page 304, where, in discussing its etiology, he uses the following language: "But that some such typhus-germ, of the fungus group, where microsphere, bacterium, or spiral formation, is the immediate cause, seems at least much more probable than any other idea."

The author assigns the birth-place of typhus to Ireland, and, of course, to be consistent with the germ theory, disputes the assertions of many Irish physicians, and of Virchow, that famine has any influence in the production of typhus. In considering the symptomatology of the disease, he asserts that petechial eruptions are present in eighty per cent. of the cases, and this fact seems to him an additional point of contact between this and the infectious exanthemata.

The sections on temperature, pulse and skin, are carefully prepared, though the discrepancy between the author's observations of temperature, and those of Wunderlich, are very striking. The affections of the nervous system are dismissed summarily, and indicate that the author has not regarded the disease very attentively from this point of view. The sections on pathological anatomy, diagnosis and prognosis, are full and complete. Concerning treatment it may be said that the author places little reliance upon drugs, but much upon prophylaxis and hygienic measures. The only drugs indicated as valuable, are diffusible stimulants and quinine, with opium and morphine incidentally.

Lebert's treatise upon Cholera comprises a reliable account of the origin, history and progress of the disease so far as the successive epidemics in Western Asia and Europe have become the scenes of its ravages. Its appearance in North America is indicated in a paragraph of ten lines, and in this particular, as in all others, the authors of the different articles in this work ignore entirely the whole field of epidemiology in America. This cannot be regarded otherwise than as a defect in a work in many respects so admirable, as from the differences in physical geography, in climate, in topography, and soil, but above all else in social conditions and surroundings, the subject could not fail to present to the student in America data of great value in the formation of correct conclusions, and which might materially change those derived from observations made in other fields exclusively under conditions differing so widely.

The author asserts his belief, unequivocally, in the efficient agency of a cholera germ, and even attempts to identify it as belonging to the "protomycetes," and "probably to those of more rounded forms," to all of which assumptions we must interpose the Scotch verdict "non probatur." We do not deny the existence of cholera germs nor of typhus germs, etc., nor the array of facts adduced in support of the germ-theory, but until the missing links essential to the completeness of the chain of argument are supplied, they must remain in the category of unclassified facts. The symptomatology of the disease is well and clearly specified, although we would have been glad to see its neuro-pathological phase a little more minutely detailed.

The subject of Prophylaxis, international and local, is fully and satisfactorily discussed, and the "general regulations to be observed upon the appearance of cholera in a locality" clearly laid down.

It is clear that the distinguished author places much more reliance upon prophylaxis than upon treatment, upon which subject his recommendations are rather negative than positive.

Lebert's great reputation must command for this portion of the work the attention of all earnest students of practical medicine.

One of the best articles in this volume is that upon Yellow Fever, by Haenisch, who has evidently studied carefully authorities upon this side of the ocean, making free use of the writings of La Roche and others. The author's analysis of the conditions under which the disease occurs and is propagated, is clear, and is sustained by the experience of observers who have had the opportunity to examine the disease in infected localities. The section upon treatment, is not so full nor so explicit as is desirable.

Heubner's article on Dysentery and that of Oertel on Diphtheria conclude this first volume of the series.

Of the first, we may say that the author seems less under the domination of preconceived ideas than are some of his colaborers, and hence is less dogmatical in his assertions. The historical portion of his article is especially interesting. Concerning treatment, this author, too, is disposed to rely largely on prophylaxis and general principles, and we are inclined to think that this disease, as it has presented itself to him, must have assumed a milder type than that which will be found to characterize it as occurring in the practice of his American readers.

Oertel, in his article upon Diphtheria, proclaims his allegiance to the germ-theory in these words, quoted from Eberth, "*without micrococci there can be no diphtheria,*" and farther, in the same paragraph, proclaims his adherence to that of spontaneous generation, in asserting that the question, "whether they (bacteria) form in the blood as does acetic acid in alcohol," . . . "in the present state of scientific knowledge must be left undetermined."

Aside from the author's assumptions regarding the etiology of disease, his treatise will well repay careful study; it is systematic, and concise, and at the same time elaborate and detailed. Its pathology, both direct and incidental, is copiously presented, and the treatment consistent and rational.

This article, on the whole, presents evidences of more extensive scientific culture and thought than any of the series.

It is impossible to give further space within our brief limits to the consideration of the great work whose examination has already occupied so much of our space belonging to other matter.

We have been somewhat elaborate in its review from the desire to call the attention of the profession generally to it, in the hope that they would study for themselves, and approve or disapprove, according to their own judgment, our criticism or praise.

The paper, typography and mechanical work of the volume are excellent and beyond criticism. H.

BOOKS RECEIVED.

OUTLINES OF THE SCIENCE AND PRACTICE OF MEDICINE. By *William Aitken, M.D., F.R.S.*, Professor of Pathology in the Army Medical School, etc., etc. London: Charles Griffin & Co. Philadelphia: J. B. Lippincott & Co. 1874.

A GUIDE TO THE PRACTICAL EXAMINATION OF URINE, for the use of Physicians and Students. By *James Tyson, M.D.*, Hospital Lecturer on Pathological Anatomy in the University of Pennsylvania, etc., etc. With a plate, and numerous illustrations. Philadelphia: Lindsay & Blakiston. 1875.

CLINICAL LECTURES ON DISEASES OF THE URINARY ORGANS. Delivered at the University College Hospital, by *Sir Henry Thompson*, Surgeon Extraordinary to his Majesty the King of the Belgians, etc., etc. Second American, from the third and revised London, Edition. Philadelphia: Henry C. Lea. 1874.

The Medical Uses of Alcohol and Stimulants for Women. By *James Edmunds, M.D.*, Member of the Royal College of Physicians of London, Member of the Royal College of Surgeons of London, etc., etc. New York: National Temperance Society and Publication House. 1874.

Cyclopædia of the Practice of Medicine. Edited by *Dr. H. Von Ziemssen*, Professor of Clinical Medicine in Munich, Bavaria. Vol. I, Acute Infectious Diseases. By Professor Liebermeister, of Tubingen, Professor Lebert, of Breslau, Dr. Haenisch, of Griefswald, Prof. Heubner, of Leipsig, and Dr. Oertel, of Munich. New York: William Wood & Co. 1874.

A Specific Diagnosis of the Study of Disease, with Special Reference to the Administration of Remedies. By *John M. Scudder, M.D.*, Professor of Pathology and Practice of Medicine in the Eclectic Medical Institute. Cincinnati: Wilstach, Baldwin & Co. 1874.

Examination of the Urine. By *George B. Fowler, M.D.*, Examiner in Physiology, College of Physicians and Surgeons, New York; Fellow of New York Academy of Medicine, etc., etc. New York: D. Appleton & Company. 1874.

Orthopædia, or a Practical Treatise on the Aberrations of the Human Form. By *James Knight, M.D.*, Member of the Medico-Chirurgical Faculty of Maryland, the District Medical Society of Ohio, and the County Medical Society of New York; Physician and Surgeon in charge of the Hospital of the New York Society for the Relief of the Ruptured and Crippled, New York City, etc., etc. New York: G. P. Putnam's Sons. 1874.

Catalogue of the Specimens in the Pathological Museum of the Philadelphia Hospital.

### PAMPHLETS RECEIVED.

The Drift of Meical Philosophy. An Essay, by *D. A. Gorton, M.D.* Philadelphia: J. B. Lippincott & Co. 1875.

Ninety-Second Annual Catalogue of the Medical School (Boston) of Harvard University. 1874 and '75.

An Account of the Epidemic of Cholera, during the Summer of 1873, in Eighteen Counties of the State of Kentucky. By *Ely McClellan, M.D.*, Asst. Surg. U. S. A. Cambridge: Printed at the Riverside Press. 1874.

ADDRESS, delivered before the American Academy of Dental Science at their Seventh Annual Meeting, held in Boston, Sept. 28, 1874. By *Dr. W. W. Allport.*

ADDRESS, delivered before McDowell Medical Society of Ken. tucky, at its Semi-Annual Meeting, in Madisonville, Ky., Nov. 4, 1874. By *W. T. Briggs, M.D.*, of Nashville, Tenn-

CLINICAL UREAMETRY. By *Henry G. Piffard, M.D.*, Surgeon of the Charity Hospital, Clinical Professor of Dermatology, Medical Department of the New York University. Reprinted from the New York Medical Journal, Dec., 1874. New York: D. Appleton & Co. 1874.

KOUMIS, AND ITS USES IN MEDICINE. By *Victor Jagielski, M.D.*, of Berlin, late Superior Physician in the Prussian Army. Chicago: Published by A. Ahrend, Chemist, 521 W. Madison street. 1874.

SEVENTH ANNUAL REPORT of the Central Free Dispensary of West Chicago, for the year ending June 30th, 1874.

REPORT of the Committee on Idiocy, made to the Illinois State Medical Society at the Annual Meeting held in Chicago, May, 1874.

JOURNALS RECEIVED.

The American Practitioner, Louisville—November, December.
The American Medical Weekly, Louisville—November 21, 28, December 5, 12, 19.
The Abstract of Medical Sciences—Vol. 1, July to Dec., 1874.
The Boston Medical and Surgical Journal—December 1.
The Boston Journal of Chemistry—December.
The Clinic, Cincinnati, Nov. 21, 28, Dec. 5, 12.
The Cincinnati Lancet and Observer—December.
The Clinical Record, Missouri—December.
The Canada Lancet—December.
The Canada Medical and Surgical Journal—December.
The Druggists' Circular—December.
The Detroit Review of Medicine and Pharmacy—December.
The Dental Cosmos—December.
Journal de l'Anatomie et de la Physiologie de l'homme et des Animaux. Charles Robin—Nov. and Dec.—No. 6.
The London Lancet—November, 1874.
The Laboratory, Boston—November.
The Medical Herald, Kansas City—Nov., Dec.
The Medical Examiner, Chicago—Nov., Dec. 1 and 15.
The Medical Record, New York—Nov., Dec. 1.
The Medical and Surgical Reporter, Philadelphia—Nov. 21, 28, Dec. 5, 12, 19.

The Medical Times, Philadelphia—Nov. 21, Dec. 5, 12, 19.
The Medical Press and Circular, London—Nov. 18, 25.
The Medical Record, New York (weekly)—Dec. 1.
The Medical News and Library and Supplement—December.
The Nashville Journal of Medicine and Surgery—Dec.
The New York Medical Journal—December.
The Ohio Medical and Surgical Reporter—November.
The Pacific Medical and Surgical Journal—Nov., Dec.
The Psychological and Medico-Legal Journal—Nov., Dec.
The Practitioner, London—Nov.
Le Progres Medical—Nos. 41, 42, 43, 44, 45.
The Physician and Pharmacist—November.
The Pharmacist, Chicago—December.
The Peninsular Journal—December.
The Richmond and Louisville Medical Journal—Nov.
Repertorio Jalisciense de Medicina y Cirurgia Practicas—Numero 1 and 2—Guadalajara. 1874.
The Sanitary Journal, Toronto—Nov.
The St. Louis Medical and Surgical Journal—Dec.
The Southern Medical Record—November.
The Technologist, New York—Nov.
The Virginia Medical Monthly—Dec.

